# Relative numerical middle in rhesus monkeys

**DOI:** 10.1098/rsbl.2021.0426

**Published:** 2022-02-09

**Authors:** Rosa Rugani, Michael L. Platt, Zhaoying Chen, Elizabeth M. Brannon

**Affiliations:** ^1^ Department of General Psychology, University of Padua, Padua, Italy; ^2^ Department of Psychology, School of Arts and Sciences, University of Pennsylvania, Philadelphia, PA, USA; ^3^ Department of Neuroscience, Perelman School of Medicine, University of Pennsylvania, Philadelphia, PA, USA; ^4^ Marketing Department, The Wharton School, University of Pennsylvania, Philadelphia, PA, USA

**Keywords:** number bisection, middle, rhesus monkeys, comparative cognition, centre, abstract concepts

## Abstract

Animals show vast numerical competence in tasks that require both ordinal and cardinal numerical representations, but few studies have addressed whether animals can identify the numerical middle in a sequence. Two rhesus monkeys (*Macaca mulatta*) learned to select the middle dot in a horizontal sequence of three dots on a touchscreen. When subsequently presented with longer sequences composed of 5, 7 or 9 items, monkeys transferred the middle rule. Accuracy decreased as the length of the sequence increased. In a second test, we presented monkeys with asymmetrical sequences composed of nine items, where the numerical and spatial middle were distinct and both monkeys selected the numerical middle over the spatial middle. Our results demonstrate that rhesus macaques can extract an abstract numerical rule to bisect a discrete set of items.

## 1. Introduction

Animals have an intuitive number sense which supports the capacity to distinguish which of two sets of objects is numerically greater [1,2], to perform simple summations and subtractions [3–6], to identify a specific ordinal position in a sequence [7–9] and to compare proportions [10,11]. While humans and animals share the approximate number system (ANS), a fundamental difference between humans and animals is that only humans are capable of precise calculations afforded by the acquisition of a counting system and symbols for number. The ANS has two behavioural signatures: the magnitude and distance effects [12,13]. The magnitude effect refers to the fact that when distance is held constant it is easier to process smaller than larger values, it is easier to discriminate 2 versus 3 than 88 versus 89 dots. The distance effect refers to the observation that as the disparity (distance) increases between two numerical sets, accuracy increases (2 versus 8 is easier than 2 versus 3). Despite the abundance of scientific evidence documenting the existence and attributes of the ANS, there are few studies that address whether animals have a ‘middle’ concept [14]. Here, we ask whether rhesus monkeys can abstract a numerical rule to identify the central item in a series of discrete items.

Empirical investigation of the ‘middleness' concept dates back to 1934, when Yerkes trained chimpanzees to identify the middle container in a sequence of three containers for a food reward. When the chimpanzees were then presented with longer sequences, comprising five, seven or nine containers, they were unable to select the middle item [15]. The failure in generalizing to longer sequences could be due to the task design, which made it difficult to open each container. Subsequent tests with containers that were easier to open showed that chimpanzees could successfully identify the middle item in a 5-item sequence [16]. A single female chimpanzee even learned to pinpoint the middle item in sequences containing up to 17 items [17,18]. This single chimpanzee also succeeded when the spacing between the items was unequal across the sequence [18]. Whether the chimpanzee used a middle strategy or instead learned to identify a specific ordinal position was unclear [19]. To differentiate these ideas, it is necessary to test transfer to sequences of different lengths.

In a recent study, rhesus monkeys learned to select the middle item in horizontal sequences of three items [14]. They transferred the middle rule to longer sequences that were new in colour and shape. Crucially, monkeys were also able to select the middle item when presented with sequences of seven items, suggesting that they did not rely on an absolute numerical strategy, which would have resulted in selecting the second item on either side. The monkeys could however have used a spatial or numerical strategy to bisect the sequences.

Here, we investigated whether rhesus monkeys can flexibly use the abstract numerical concept of middle to navigate novel and expanded sequences, in a highly-controlled computerized setting. The main goal of this study was to disentangle if monkeys relied on numerical or spatial information when identifying the middle item.

## 2. Methods

### (a) Subjects

The subjects were two socially housed male rhesus macaques (*Macaca mulatta)*, named Arrow (5 years old) and Tolman (6 years old). Monkeys were separated for in-cage testing.

### (b) Apparatus

This consisted of a 15 inch touch-sensitive computer monitor (Elo TouchSystems, Menlo Park, CA) connected with a food pellet reward delivery system (Med Associates, St Albans, VT). The monitor was fixed to the front of the macaque's home cage and the pellet reward was connected with a food container behind the monitor. A program written in PsychoPy3^40^ presented the stimuli, controlled the reward delivery and collected data.

**Figure 1.**
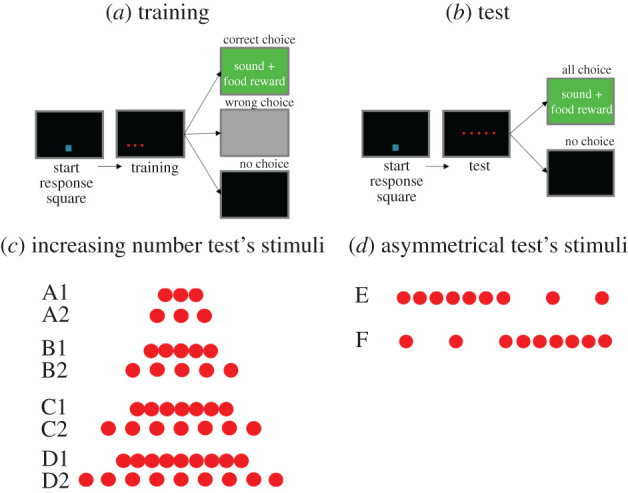
Schematic illustration of the experimental procedure and stimuli. (*a*) Training trials. A start response square then a three-dot stimulus appeared. A food reward, a green screen and a positive sound occurred after the selection of the middle dot. A grey screen appeared after the choice of either lateral dot; the screen turned black after 5 s with no choice. (*b*) Testing trials. A start response square then a stimulus appeared. The selection of all dots, lateral or middle, elicited a positive reward. The screen turned black after no choice within 5 s. (*c*) Stimuli used in the number transfer test: close 3-dots (A1), far 3-dots (A2), close 5-dots (B1), far 5-dots (B2), close 7-dots (C1), far 7-dots (C2), close 9-dots (D1) and far 9-dots (D2). (*d*) Stimuli used in the asymmetrical test: asymmetrical left condition (E) and asymmetrical right condition (F) (https://www.nature.com/articles/s41598-020-74533-8).

We first trained monkeys to select the middle dot in an array of three identical dots. To prevent monkeys from learning to touch a specific location on the screen, we presented the three-dot array at 32 different absolute positions on the screen, balanced for left/right, up/down, and we used two inter-dot distances (0.75 and 2 cm). On each training trial, monkeys earned a positive reward by touching the middle dot (figure 1*a*). We then tested the monkeys with two transfer experiments; figure 1*b* schematically represents the experimental procedure. In the number transfer test, we explored whether monkeys' performances showed a magnitude effect, which is a characteristic signature of the ANS. Specifically, we tested monkeys with sequences of 3, 5, 7 or 9 identical dots (figure 1*c*). If middle identification relies on numerical cues, responses would become less accurate as the number of dots increases. In the asymmetrical test, we attempted to disentangle whether monkeys used a numerical or spatial strategy by presenting spatially asymmetric sequences where the spatial middle and numerical middle were not the same item. Monkeys were presented with 9-item sequences in non-differentially rewarded trials (figure 1*d*).

## 3. Results

We conducted Bayes factor (BF) analyses using version 0.9.12-4.2 of the BF package in R and using the default parameter values for JASP 0.11.1. We used the classification by Lee & Wagenmakers [20] to interpret BFs. We conducted frequentist analyses using the stats package in R and JASP 0.11.1.

### (a) Number transfer test

**Figure 2.**
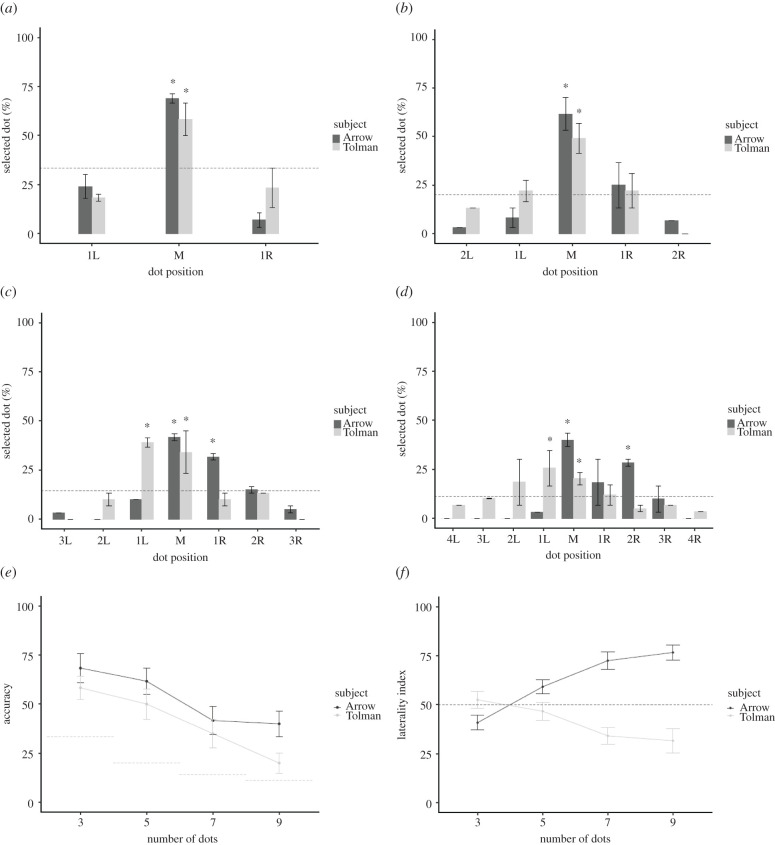
Results of the number transfer test. Monkeys primarily selected the middle dot in all sequences ((*a*) 3 items, (*b*) 5 items, (*c*) 7 items, (*d*) 9 items), and (*e*) accuracy diminished as the number of items increased. (*f*) Laterality index. Both monkeys showed a side effect in the function of numerical magnitude: Arrow showed a right bias while Tolman showed a left bias. In all graphs, the black bars represent the standard errors and the dashed line indicates chance level, and the asterisks indicate *p* < 0.05.

Performance did not differ on close and far trials (all *p* > 0.05 and BF values ranging from 0.216 to 1.731, see electronic supplementary material), leading us to merge trial types for subsequent analyses. BF analyses revealed that both monkeys transferred the middle concept from the 3-item sequence to the novel numerical sequences with above chance expectations (table 1 and figure 2*a*–*d*).

**Table 1.** Data and results concerning the selection of the middle dot for each sequence composed of 3, 5, 7 or 9 dots for each monkey, in the number transfer test. Both monkeys transferred the middle rule to longer sequences.

monkey	no. dots	no. success	no. trials	*p*	Cohen's *h*	BF
Arrow	3	40	58	<0.001	0.729	>100
	5	37	60	<0.001	0.879	>100
	7	25	60	<0.001	0.628	>100
	9	24	60	<0.001	0.690	>100
Tolman	3	35	60	<0.001	0.508	>100
	5	29	59	<0.001	0.627	>100
	7	20	59	<0.001	0.468	>100
	9	12	59	<0.050	0.256	2.839

To test whether the monkeys' accuracy decreased with magnitude, we compared accuracy across trials of different numerical lengths. BF analyses revealed strong evidence in favour of a magnitude effect for both monkeys. As shown in figure 2*e*, accuracy diminished as the number of items increased: Arrow: $\chi_{3}^{2}=11.817$, *p* = 0.008, *ε*^2^ = 0.099; BF_10_ = 10.299; Tolman: $\chi_{3}^{2}=18.013$, *p* < 0.001, *ε*^2^ = 0.151; BF_10_ = 192.092; Kruskal–Wallis rank sum test and Bayesian repeated measure ANOVA (see also figure S1 for a graphical representation of the number of errors performed on the number transfer test). The BF indicates that the data are 10.299 times (for Arrow) and 192.092 times (for Tolman) more likely under the model that includes numerical magnitude as a predictor, compared with the null model.

We next investigated whether monkeys showed a laterality effect and whether any such effect interacted with magnitude. We calculated a laterality index as the percentage of right-sided choices on the overall number of wrong choices. A laterality index of 100 would indicate that all wrong choices were to the right of the middle whereas a laterality index of −100 would indicate that all wrong choices were to the left. A laterality index of 0 would indicate that incorrect choices were equally likely on the left and right. Figure 2*f* shows that both monkeys exhibited a laterality bias that increased with the sequence length. Arrow was more likely to make rightward errors ($\chi_{3}^{2}=37.169$, *p* < 0.001, *ε*^2^ = 0.312; BF_10_ > 100), whereas Tolman was more likely to make leftward errors ($\chi_{3}^{2}=13.047$, *p* =0.004, *ε*^2^ = 0.11; BF_10_ = 8.568) as the number of dots increased.

### (b) Asymmetrical test

Performance did not differ for leftward and rightward asymmetric arrays for either monkey as indicated by the null evidence provided by the BF (Arrow: $\chi_{8}^{2}=7.915$, *p* = 0.442, phi = 0.297; BF_10_ = 0.038; Tolman: $\chi_{8}^{2}=8.451$, *p* = 0.395, *φ* =0.306; BF_10_ = 0.313; Pearson's chi-squared test and Bayesian contingency tables; electronic supplementary material, figure S2 depicts the distribution of the responses). One-tailed exact binomial tests were used to establish if the spatial middle and numerical middle items were selected with above-chance expectations on each trial type. Monkeys selected the numerical middle, but not the spatial middle, with above-chance expectation. BF analysis yielded extreme and strong evidence in favour of numerical middle identification, for Arrow and Tolman respectively, and null evidence for spatial identification (Arrow, numerical middle: number of successes = 20, number of trials = 72, *p* < 0.001, Cohen's *h* = 0.434; BF_10_ > 100; spatial middle: number of successes = 1, number of trials = 72, *p* = 0.999, Cohen's *h* = −0.440; BF_10_ = 0.015; Tolman, numerical middle: number of successes = 17, number of trials = 72, *p* < 0.001, Cohen's *h* = 0.339; BF_10_ = 13.63; spatial middle number of successes = 6, number of trials = 72, *p* = 0.817, Cohen's *h* = −0.090; BF_10_ = 0.036; exact binomial test and Bayesian binomial test). Both monkeys showed strong evidence for the selection of the numerical middle over the spatial middle item on each trial type (Arrow, numerical middle: mean = 27.778, s.e. = 4.648; spatial middle: mean = 1.388, s.e. = 1.388; *W* = 21, *p* = 0.017, *r* = 1; BF_10_ = 28.505; Tolman, numerical middle: mean = 23.612, s.e. = 1.388; spatial middle: mean = 8.333, s.e. = 3.044; *W* = 21, *p* = 0.017, *r* = 1; BF_10_ = 25.623; paired Wilcoxon test and Bayesian Wilcoxon signed-rank test).

## 4. Discussion

A plethora of experimental research demonstrates animal numerical competence [3–5]. Here, we investigated whether rhesus monkeys spontaneously extract a numerical ‘middle’ concept when they are trained to identify the numerical and spatial middle of a sequence of 3 discrete items. Monkeys preferentially chose the middle item in novel sequences of 3, 5, 7 and 9 items. Monkeys further showed a magnitude effect, with accuracy that decreased as the number of items increased. Although number and space were confounded in our first transfer test to novel numerical values, we dissociated the two with an asymmetric transfer test. We presented monkeys with asymmetrical sequences where the numerical middle item was either on the left or on the right side with respect to the spatial centre. Monkeys preferentially chose the numerical middle and ignored the spatial centre, providing strong support that they spontaneously encoded the numerical middle concept.

In prior research using a manual line bisection task, symbolic and non-symbolic number has been shown to bias bisection even though the numerical exposure was task-irrelevant [21,22]. In those studies adults, young school children, and preschool children were instructed to indicate the midpoint of a horizontal line that was flanked by two arrays of dots of unequal values. Non-symbolic numerical displays systematically biased localization of the midpoint, toward the display depicting the larger magnitude, at all ages. Numerical information was thus automatically extracted from visual arrays of dots, even though number was irrelevant. That phenomenon testifies to the close relationship between spatial and numerical representations [22]. In our task, even though monkeys could have exploited spatial cues to learn the training task, they relied on numerical but not spatial cues when faced with a transfer test in which they could have used either. Our findings are consistent with a previous study with chicks in which numerical and spatial information were redundant during training and dissociated at test. In that study, day-old chicks learned to peck the fourth container in a series of 10 identical ones. When, at test, they faced a smaller number of containers, five, and a conflict between ordinal and spatial cue, chicks selected only the numerically correct container [23]. Numerical information appears to be very salient for animals and automatically processed even in circumstances in which other cues could drive behaviour.

Dehaene *et al.* [24] first demonstrated the SNARC (spatial numerical association of response code) effect, providing strong empirical evidence that humans represent numbers on a mental number line, usually oriented from left to right. Subsequent work with pre-linguistic children, infants, newborns and non-human animals suggests that spatial representation of number emerges early in human ontogeny and it is shared by different species [25–27].

We found that the numerosity of a sequence affected middle identification biasing errors. One monkey's errors became increasingly right-biased and the other monkey's errors became increasingly left-biased with sequence length. This was consistent with the distribution of choices, which was characterized by significant errors to the right of middle for Arrow and to the left of middle for Tolman. This lateral bias was not evident on the asymmetrical test, possibly because of the unbalanced displacement of the items in the series. The two monkeys may have anchored to the left and right and scanned the environment from either side. This finding suggests that the mapping of number onto space may be more flexible in monkeys than humans and show strong individual differences. This pattern of results is consistent with recent evidence in adult gorillas, orangutans and birds [28,29]. Despite variability in the individual directionality of the spatial numerical association (SNA), its presence in most subjects suggests that mapping number onto space may be a widespread cognitive strategy. Idiosyncratic experiences may influence the individual orientation of the spatial numerical association.

The present study provides strong evidence that supports our previous finding that monkeys can identify the middle in sequences of discrete items and extends the findings in two important ways [14]. First, we show that monkeys transfer a middle rule learned with a small set of discrete items to a larger set of discrete items. Second, we demonstrate that, despite having learned the middle rule with sequences for which spatial and numerical cues were confounded, monkeys abstracted numerical information only. Middle identification should thus be considered part of the suite of quantitative abilities supported by the approximate number system.

## Data accessibility

Our datasets, data and codes are stored and backed up on the Research Data Unipd server: http://researchdata.cab.unipd.it/527/ doi:10.25430/researchdata.cab.unipd.it.00000527. The data are provided in the electronic supplementary material.
